# Ursodeoxycholic acid suppresses the malignant progression of colorectal cancer through TGR5-YAP axis

**DOI:** 10.1038/s41420-021-00589-8

**Published:** 2021-08-07

**Authors:** Huan Zhang, Huanji Xu, Chenliang Zhang, Qiulin Tang, Feng Bi

**Affiliations:** Department of Medical Oncology, Cancer Center and Laboratory of Molecular Targeted Therapy in Oncology, West China Hospital, Sichuan University, Chengdu, Sichuan Province China

**Keywords:** Colon cancer, Prognostic markers, Cancer prevention, Cancer prevention

## Abstract

The Hippo/YAP pathway plays an important role in the development of cancers. Previous studies have reported that bile acids can activate YAP (Yes Associated Protein) to promote tumorigenesis and tumor progression. Ursodeoxycholic acid (UDCA) is a long-established old drug used for cholestasis treatment. So far, the effect of UDCA on YAP signaling in colorectal cancer (CRC) is not well defined. This study means to explore relationship of UDCA and YAP in CRC. UDCA suppressed YAP signaling by activating the membrane G-protein-coupled bile acid receptor (TGR5). TGR5 mainly regulated cAMP/PKA signaling pathway to inhibit RhoA activity, thereby suppressing YAP signaling. Moreover, the restoration of YAP expression alleviated the inhibitory effect of UDCA on CRC cell proliferation. In AOM/DSS-induced CRC model, UDCA inhibited tumor growth in a concentration-dependent manner and decreased expression of YAP and Ki67. UDCA plays a distinguished role in regulating YAP signaling and CRC growth from the primary bile acids and partial secondary bile acids, demonstrating the importance of maintaining normal intestinal bile acid metabolism in cancer patients. It also presents a potential therapeutic intervention for CRC.

## Introduction

The Hippo/YAP pathway regulates organ size, tumor formation, and function of stem cells. Excessive activation of YAP has been implicated in a variety of human malignant tumors including colorectal cancer (CRC) [1]. YAP activation also plays a critical role in the resistance to EGFR-MAPK targeted drugs or other therapies [2–4], and is generally associated with poor survival in many tumors [5, 6].

Bile acids (BAs) are the main metabolites of cholesterol, playing a major role in lipid absorption in the intestines, glucose regulation, and energy homeostasis [7]. The two primary BAs, cholic acid (CA) and chenodeoxycholic acid (CDCA), are synthesized from cholesterol in the liver, and then secreted into the intestines as taurine or glycine conjugated form. In the intestines, the conjugated BAs are transformed to secondary BAs, including lithocholic acid (LCA), deoxycholic acid (DCA), and ursodeoxycholic acid (UDCA) by gut microbiota [8, 9]. Since they are important signaling molecules, BAs modulate many physiological and pathological processes mainly through farnesoid X receptor (FXR) and the G-protein receptor 5 (TGR5), a transmembrane G protein-coupled bile acid receptor [10].

As the mainly materials to synthesis BAs, cholesterol had exerted tumor-promoting effects in many tumors and regulated mutually with Hippo/YAP pathway. Cholesterol activates the Wnt/PCP-YAP signaling in SOAT1-targeted treatment of colon cancer, resulting in the progression of CRC [11]. Mevalonic acid is the main molecular in cholesterol synthesis, and it can also promote progression of breast cancer [12]. Previous studies have reported that the primary BAs, and some secondary bile acids such as DCA and LCA, are promoters of tumorigenesis of various cancers including hepatocellular carcinoma and CRC [13, 14]. The primary BAs can activate YAP to promote liver carcinogenesis through IQGAP1 induction [15]. In addition, the secondary bile acid taurodeoxycholic acid (TDCA) can activate YAP via the vitamin D receptor (VDR) to promote melanoma metastasis [16].

The UDCA, derived from the biotransformation of CDCA by intestinal microflora, is a well-established drug approved for the treatment of primary biliary cholangitis (PBC) and primary sclerosing cholangitis (PSC), with a good safety profile and minimal side effects even used at high doses [17, 18]. Several studies have reported that UDCA exert anti-tumor properties in multiple tumors including melanoma, hepatocellular carcinoma, and colorectal cancer [19–21]. However, the preventive effect of UDCA on CRC has been challenged in recent years [22]. The effect of UDAC on YAP signaling and its implication in CRC has not been elucidated despite UDCA sharing the same receptors with the primary BAs and some secondary bile acids.

The results obtained in this study indicated that UDAC suppressed YAP signaling by activating the TGR5-cAMP-PKA axis, thereby decreasing RhoA activity and YAP signaling. Moreover, overexpression of YAP alleviated the inhibitory effect of UDAC on CRC cell proliferation. The UDAC prevented tumor growth in a dose-dependent manner by decreasing YAP expression in vivo.

These findings show that UDCA and primary BAs or some secondary bile acids have opposite effects on YAP signaling and CRC growth, indicating the importance of maintaining normal intestinal bile acid metabolism in cancer patients. In addition, UDCA supplementation may confer potential therapeutic effects in CRC with high TGR5 expression.

## Results

### UDCA suppressed the expression of YAP and its target genes

**Fig. 1:**
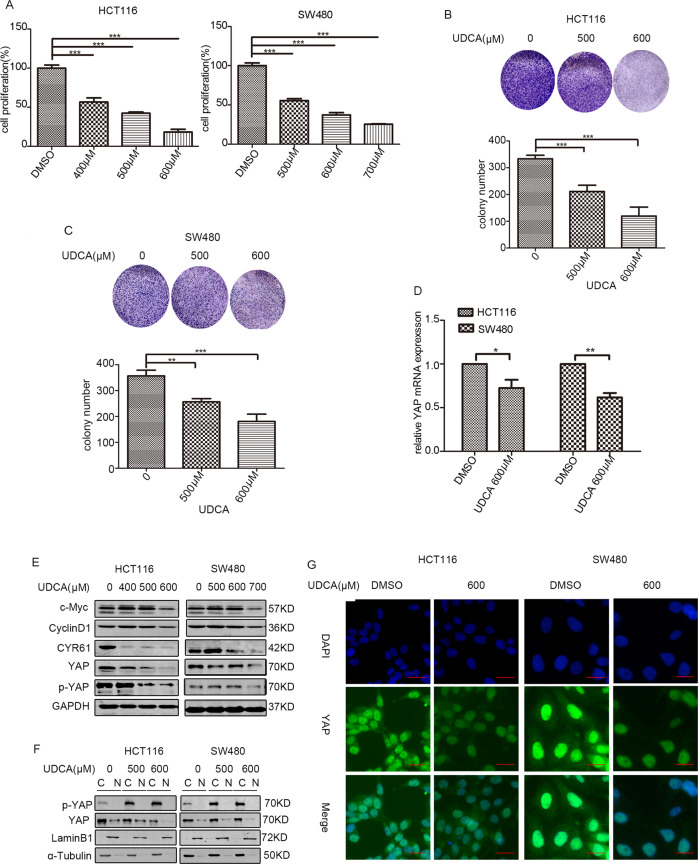
UDCA regulates expression of YAP and Hippo pathway target genes. **A** UDCA-induced inhibition of cell proliferation was measured using MTT assays after treatment for 36 h. Data are demonstrated as the mean ± SD, *p < 0.05, **p < 0.01, and ***p < 0.001 with Student’s *t* test (two-tailed). **B**, **C** Clonogenic assays and qualitative analysis of the HCT116 and SW480 cells cultured with the indicated concentration of UDCA at day 14. **D** YAP mRNA levels in HCT116 and SW480 cells were measured with quantitative real-time RT-PCR, and normalized to GAPDH. Data are demonstrated as the mean ± SD, *p < 0.05, **p < 0.01, and ***p < 0.001 with Student’s *t* test (two-tailed). **E** WB for c-Myc, cyclinD1, p-YAP, YAP, and CYR61 in the HCT116 and SW480 cells treated with UDCA for 36 h. **F** WB results showing YAP expression in cytoplasmic and nuclear protein extracts from HCT116 and SW480 cells treated with DMSO or UDCA (500 μM and 600 μM) for 36 h. **G** HCT116 and SW480 cells were treated with 600 μM UDCA for 36 h, and YAP expression was visualized by IF staining with anti-YAP antibody (green). DNA was stained using DAPI (blue). Scale bar: 25 μm.

MTT assays showed that UDCA inhibited the proliferation of HCT116 and SW480 cells in a concentration-dependent manner (Fig. 1A). Results of the colony formation assays also demonstrated that UDCA suppressed the survival of HCT116 and SW480 cells (Fig. 1B-C). Based on qPCR assays, we found that UDCA diminished the expression of YAP mRNA in HCT116 cells and SW480 cells (Fig. 1D). Further Western blot (WB) assays also revealed that UDCA decreased the expression of YAP and CYR61 in a concentration-dependent manner, while increased ration of p-YAP to YAP (Figs. 1E and S1A). The nuclear protein detection assays and IF assays further showed that UDCA decreased the expression levels of YAP in the nucleus, and inhibited activity of YAP (Fig.1F, G). These data indicated that UDCA inhibited the expression of YAP and its target gene in CRC cells.

### UDCA inhibited HCT116 cells and SW480 cells survival through YAP signaling

**Fig. 2:**
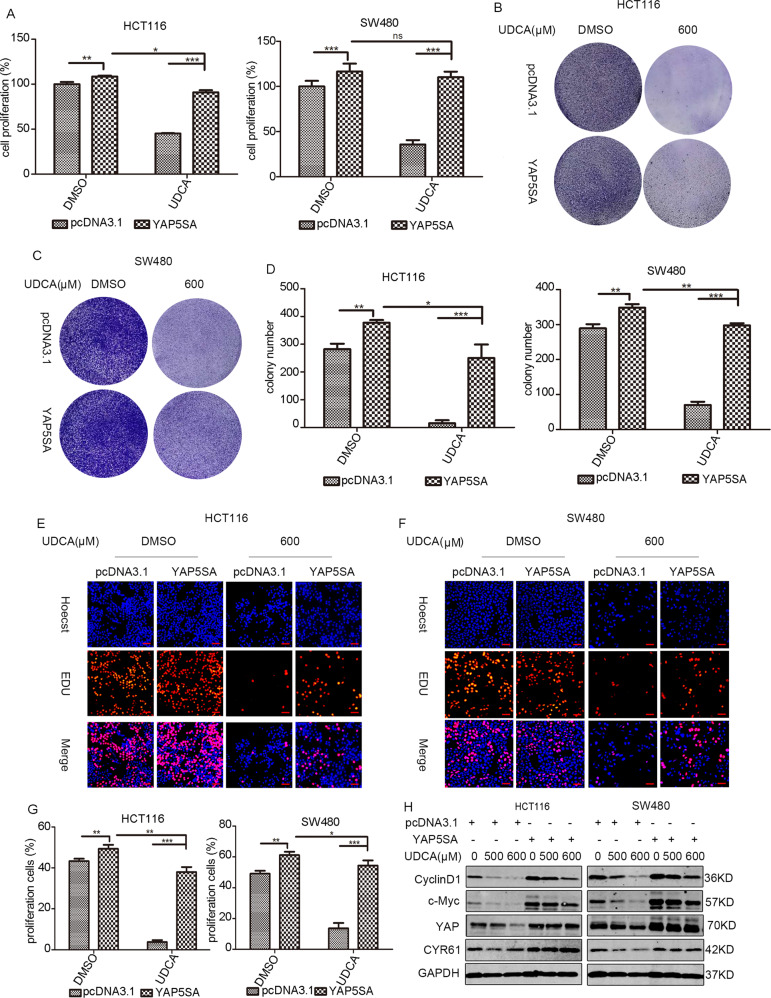
UDCA regulates the survival of HCT116 cells and SW480 cells through the Hippo pathway. **A** MTT assay for HCT116 and SW480 cells cultured with pcDNA3.1 or YAP5SA plasmid (or/and 500 μM or 600 μM UDCA in HCT116 and SW480 cells, respectively) for 3 days. Data are demonstrated as the mean ± SD, *p < 0.05, **p < 0.01, and ***p < 0.001 with Student’s *t* test (two-tailed). **B**–**D** Clonogenic assays and qualitative analysis of the HCT116 and SW480 cells cultured with pcDNA3.1 or YAP5SA plasmid (or/and 600 μM UDCA) at day 7. EdU incorporation assay was performed to further test the cell proliferation at 72 h post-transfection in HCT116 cells (**E**) and SW480 cells (**F**). **G** Red labeled cells indicated the proliferated cells. The percentage of the proliferated cells was also counted. Data are demonstrated as the mean ± SD, *p < 0.05, **p < 0.01, and ***p < 0.001 with Student’s *t* test (two-tailed). Scale bar: 25 μm. **H** WB for CyclinD1, c-MYC, YAP, and CYR61 in HCT116 and SW480 cells cultured with pcDNA3.1 or YAP5SA plasmid (or/and 500 μM or 600 μM UDCA in HCT116 and SW480 cells, respectively) for 3 days.

YAP plasmids were then transfected into HCT116 and SW480 cells to further explore whether UDCA acts through YAP to inhibit the survival of CRC cells. The cell proliferation assays and colony formation assays showed that overexpression of YAP can partially reverse the cell proliferation inhibition induced by UDCA treatment in HCT116 and SW480 cells (Fig. 2A–D). The above results were confirmed by EdU cell proliferation assays (Fig. 2E–G). Immunoblotting tests further demonstrated that YAP overexpression reversed the effects of UDCA on the expression of c-Myc and cyclin D1, two critical proteins associated with cell proliferation in HCT116 and SW480 cells (Fig. 2H).

We have performed YAP silencing experiments with small interfering RNAs (siRNAs). However, we found that UDCA can still exert inhibitory effect on CRC cell lines in case of YAP knock-down, even exert more powerful inhibition effect on CRC cells proliferation. This confusing results may due to that siYAP used in our experiments can only partially silence YAP expression, and when combined with UDCA, YAP activity was suppressed synergistically.

To further conform the role of YAP on CRC cells proliferation, we have performed YAP silencing experiments with siRNAs. However, we found that UDCA can still exert inhibitory effect on CRC cell lines in case of YAP knock-down, even exert more powerful inhibition effect on CRC cells proliferation (Fig. S2). These confusing results may be due to that siYAP RNAs used in our experiments that can only partially silence YAP expression, and when combined with UDCA, YAP activity was suppressed synergistically. Together, these results indicated that UDCA inhibited the proliferation of cells by inhibiting the YAP pathway in CRC.

### UDCA inhibited YAP pathway by suppressing RhoA activity

**Fig. 3:**
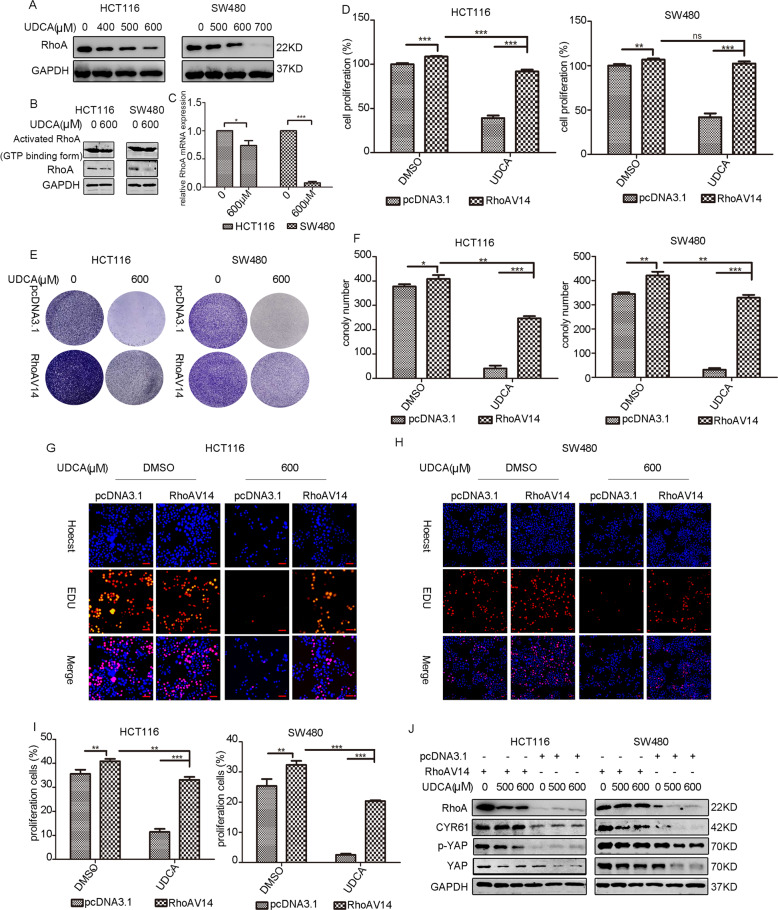
UDCA inhibits Hippo pathway through the downregulation of RhoA expression. **A** WB for RhoA in HCT116 and SW480 cells treated with DMSO or UDCA (400 μM, 500 μM, and 600 μM in HCT116 cells; 500 μM, 600 μM, and 700 μM in SW480 cells) for 36 h. **B** Active RhoA detected by the RhoA pull down assay in HCT116 and SW480 cells treated with DMSO or 600 μM UDCA for 36 h. **C** Quantitative real-time RT-PCR was used to measure the RhoA mRNA level in HCT116 and SW480 cells. GAPDH was used as a control, and ***p < 0.001 using Student’s *t* test (two-tailed). **D** MTT assay for HCT116 and SW480 cells cultured with pcDNA3.1 or RhoAV14 plasmid (or/and 600 μM UDCA) for 3 days. Data are demonstrated as the mean ± SD, *p < 0.05, **p < 0.01, and ***p < 0.001 with Student’s *t* test (two-tailed). **E**, **F** Clonogenic assays and qualitative analysis of the HCT116 and SW480 cells cultured with pcDNA3.1 or RhoAV14 plasmid (or/and 600 μM UDCA) at day 7. EdU incorporation assay was performed to further test the cell proliferation at 72 h post-transfection in HCT116 cells (**G**) and SW480 cells (**H**). Scale bar: 25 μm. Red labeled cells indicated as the proliferated cells. **I** The percentage of the proliferated HCT116 cells and SW480 cells. Data are demonstrated as the mean ± SD, *p < 0.05, **p < 0.01, and ***p < 0.001 with Student’s *t* test (two-tailed). **J** WB for RhoA, YAP, p-YAP, and CYR61 in HCT116 and SW480 cells cultured with pcDNA3.1 or RhoAV14 plasmid (or/and 500 μM and 600 μM UDCA).

A previous study reported that RhoA regulates the activation of YAP/TAZ [23]. Herein, WB assay results indicated that UDCA decreased the expression of RhoA in CRC cells (Fig. 3A). UDCA also decreased the expression level of total RhoA and active RhoA as revealed by the GST-pull down assay (Fig. 3B). In addition, we observed an increase of p-MYPT expression levels and a downregulation of the p-MYPT/MYPT ratio, suggesting diminished RhoA activation (Fig. S3A–B). qPCR assay further revealed that UDCA treatment downregulated RhoA expression at the mRNA level (Fig. 3C). RhoA plays a critical role in the formation of F-actin stress fibers, which is important for cell morphology and assembly of focal adhesion (FA) linking the actin cytoskeleton to the extracellular matrix [24, 25]. Therefore, the cells were stained using rhodamine-labeled phalloidin to observe the cell morphology. Results showed that UDCA impaired stress fiber formation leading to abnormal cell morphology (Fig. S3C). Cell proliferation assays showed that RhoA overexpression reversed the inhibitory effect of UDCA treatment on cell survival in HCT116 cells and SW480 cells (Fig. 3D–I). Overexpression of RhoA also reversed the UDCA-induced downregulation of YAP expression in HCT116 cells and SW480 cells (Figs. 3J and S1B). These results revealed that UDCA acted through RhoA to inhibit the YAP pathway in CRC cells.

### UDCA stimulated cAMP-PKA-RhoA pathway to inhibit YAP

**Fig. 4:**
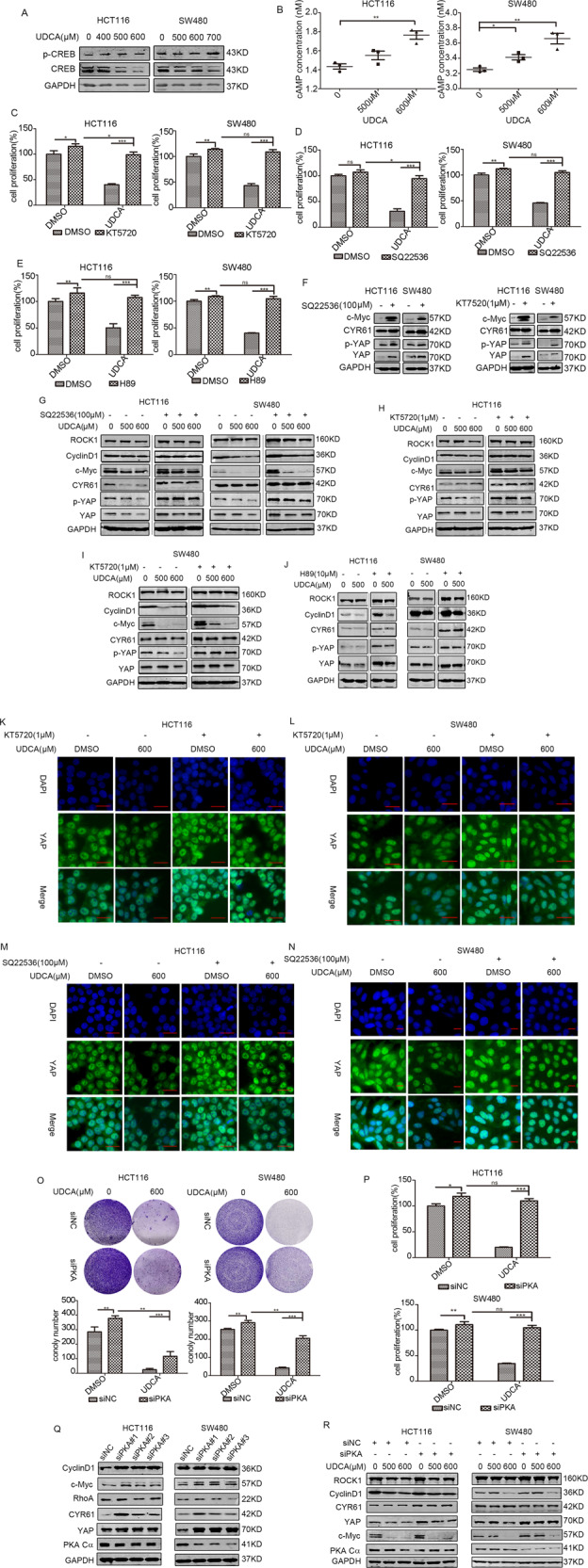
UDCA downregulates Hippo pathway by stimulating the cAMP-PKA pathway. **A** WB for CREB and p-CREB in HCT116 and SW480 cells cultured with DMSO or UDCA (400 μM, 500 μM, and 600 μM in HCT116 cells; 500 μM, 600 μM, and 700 μM in SW480 cells) for 36 h. **B** cAMP levels were measured with ELISA assay. HCT116 cells and SW480 cells were pretreated with 1 μM KT5720 for 30 min (**C**), 100 μM SQ22536 for 30 min (**D**) or 10 μM H89 for 24 h (**E**), and then HCT116 and SW480 cells were treated with 500 μM or 600 μM UDCA for 36 h. Cell viability was measured using the MTT assay. Data are demonstrated as the mean ± SD, *p < 0.05, **p < 0.01, and ***p < 0.001 with Student’s *t* test (two-tailed). **F** WB for c-Myc, CYR61, YAP, and p-YAP in HCT116 and SW480 cells treated with 1 μM KT5720 or 100 μM SQ22536 for 30 min. **G**–**J** Immunoblotting assay in HCT116 and SW480 cells pretreated with 1 μM KT5720, 100 μM SQ22536 for 30 min, or 10 μM H89 for 24 h, and then treated with 500 μM or 600 μM UDCA for 36. **K**–**N** The HCT116 cells and SW480 cells were pretreated with KT5720, SQ22536, or H89, and then they were treated with 600 μM UDCA. YAP expression was visualized using IF staining with anti-YAP antibody (green). DNA was stained using DAPI (blue). Scale bar: 25 μm. **O** Clonogenic assays and qualitative analysis of the HCT116 and SW480 cells cultured with the negative control or siTGR5 (or/and 600 μM UDCA) at day 7. **P** MTT assay for HCT116 and SW480 cells cultured with the negative control or siPKA (or/and 600 μM UDCA) for 3 days. **Q** WB for PKA Cα, CyclinD1, c-Myc, YAP, CYR61, and RhoA in HCT116 and SW480 cells transfected with siPKA or the negative control for 3 days. **R** WB for PKA Cα, c-Myc, YAP, and CYR61 in HCT116 and SW480 cells cultured with the negative control or siTGR5 (or/and 500 μM, 600 μM UDCA) for 3 days.

Previous studies have reported that the activation of cAMP/PKA pathway could suppress YAP in the condition of actin cytoskeletal changes, and cAMP/PKA-mediated inhibition of RhoA/ROCK played a critical role in the regulation of vascular endothelium contractile [26, 27]. Results of WB assay indicated that UDCA treatment increased p-CREB expression, a direct target of PKA, indicating PKA activation and cAMP accumulation (Fig. 4A). On the other hand, ELISA assays showed upregulation of cAMP levels in HCT116 and SW480 cells following treatment with the indicated concentrations of UDCA (Fig. 4B). In addition, pharmacological blockade of the cAMP/PKA pathway reversed the inhibitory effect of UDCA on cell growth (Fig. 4C–E). Further WB and IF results confirmed that inhibition of cAMP/PKA pathway reversed UDCA-mediated downregulation of the YAP pathway (Figs. 4F–N and S1C–F). Similarly, siRNA-mediated silencing of PKA partially reversed UDCA-induced inhibition on cell growth inhibition and downregulation of YAP (Fig. 4O–R). Collectively, these experimental results indicated that UDCA inhibited the RhoA/YAP pathway by activating cAMP/PKA signaling.

### TGR5 contributed to the regulatory effects of UDCA on the cAMP/PKA-RhoA-YAP axis

**Fig. 5:**
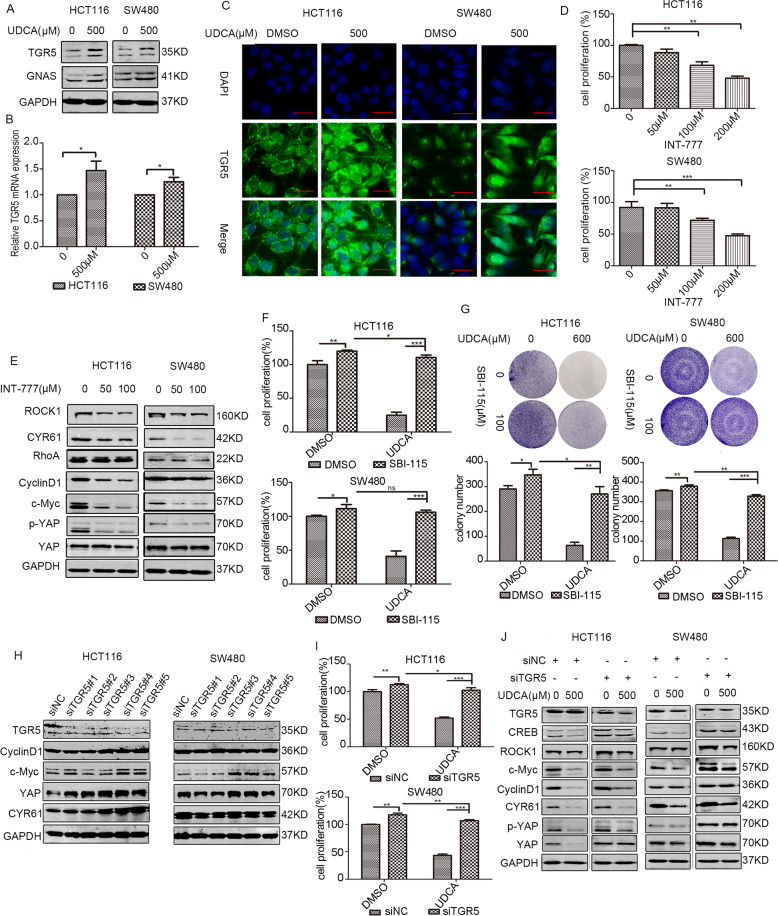
TGR5 participates in the UDCA-mediated regulation of cAMP/PKA-RhoA-YAP pathway. **A** WB for TGR5 and GNAS in HCT116 and SW480 cells after treatment with DMSO or 500 μM for 36 h. **B** Quantitative real-time RT-PCR to measure the TGR5 mRNA level in HCT116 and SW480 cells. GAPDH was used as a control, and ***p < 0.001 using Student’s *t* test (two-tailed). **C** HCT116 and SW480 cells were treated with 500 μM UDCA for 36 h. TGR5 expression was visualized using IF staining with the anti-TGR5 antibody (green). DNA was stained using DAPI (blue). Scale bar: 25 μm. **D** Cell viability of HCT116 and SW480 cells cultured with DMSO or 50 μM, 100 μM, and 200 μM TGR5 agonist INT-777 for 24 h. **E** WB for RhoA, ROCK1, CYR61, CyclinD1, c-Myc, YAP, and p-YAP in HCT116 and SW480 cells treated with DMSO and INT-777 (50 μM and 100 μM, respectively) for 24 h. **F** HCT116 and SW480 cells were pretreated with 100 μM TGR5 antagonist SBI-115 for 24 h, and then treated with the indicated concentration of UDCA for 36 h. Cell proliferation was measured using MTT assays. **G** Clonogenic assays and qualitative analysis of the HCT116 and SW480 cells pretreated with 100 μM TGR5 antagonist SBI-115 for 24 h, and then they were treated with 600 μM UDCA at day 7. Data are demonstrated as the mean ± SD, *p < 0.05, **p < 0.01, and ***p < 0.001 with Student’s *t* test (two-tailed). **H** WB for TGR5, CyclinD1, c-Myc, YAP, and CYR61 in HCT116 and SW480 cells transfected with siTGR5 or the negative control for 3 days. **I** MTT assay for HCT116 and SW480 cells cultured with the negative control or siTGR5 (or/and 600 μM UDCA) for 3 days. Data are demonstrated as the mean ± SD, *p < 0.05, **p < 0.01, and ***p < 0.001 with Student’s *t* test (two-tailed). **J** WB for TGR5, CREB, ROCK, CyclinD1, c-Myc, YAP, p-YAP, and CYR61 in HCT116 and SW480 cells cultured with the negative control or siTGR5 (or/and 500 μM UDCA) for 3 days.

TGR5, a bile acid receptor, has been previously reported to activate PKA signaling by increasing intracellular cAMP levels, and activation of TGR5 receptors can relive diabetic retinopathy by inhibiting the RhoA/ROCK pathway [28, 29]. Our results showed that UDCA treatment increased the expression of TGR5 at the mRNA and protein levels (Fig. 5A, B). IF assays revealed that UDCA not only upregulated membrane TGR5 expression, but also increased cytoplasmic TGR5 levels in HCT116 and SW480 cells (Fig. 5C). A specific TGR5 agonist, INT-777, exerted similar effects as UDCA on the proliferation of HCT116 and SW480 cells, and INT-777 also suppressed activity of RhoA-YAP aixs and inhibited expression of cell proliferation-related proteins (Figs. 5D, E and S1G). In addition, pharmacological inhibition of TGR5 abolished UDCA-induced inhibition on cell growth (Fig. 5F, G). Genetic silencing of TGR5 reversed UDCA-induced downregulation of YAP and inhibition of cell proliferation (Fig. 5H–J and S1H). Thus, UDCA can regulate YAP signaling and cell proliferation in CRC by activating TGR5.

### UDCA inhibited CRC tumor growth in vivo

**Fig. 6:**
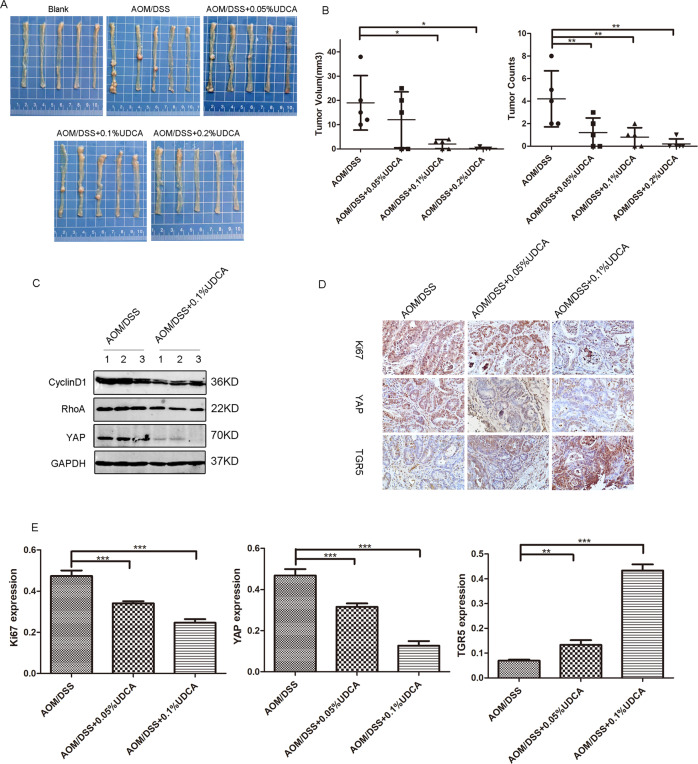
UDCA inhibited CRC tumor growth in vivo. **A** Representative gross images of AOM/DSS-induced colorectal cancer model treated with a diet containing 0, 0.05% UDCA, 0.1% UDCA, and 0.2% UDCA, respectively. **B** Tumor volume and tumor count in AOM/DSS model. Data are demonstrated as the mean ± SD, *p < 0.05, **p < 0.01, and ***p < 0.001 with Student’s *t* test (two-tailed). **C** WB for YAP, cyclinD1, and RhoA in tumor lysates from the AOM/DSS group and AOM/DSS+0.1% UDCA group. **D** Representative immunohistochemical staining images of Ki67, YAP, and TGR5 in AOM/DSS-induced tumor tissues. **E** Qualitative analysis of immunohistochemical staining. Data are demonstrated as the mean ± SD, *p < 0.05, **p < 0.01, and ***p < 0.001 with Student’s *t* test (two-tailed).

The AOM/DSS-induced primary CRC mice model was constructed to explore the effect of UDCA on the tumorigenesis and growth of CRC cells in vivo (Fig. S4D). At the indicated time points, we observed that UDCA reduced the number of tumors and tumor volumes in a dose-dependent manner in groups treated with UDCA when compared with the control group (Fig. 6A, B). Mechanistic studies indicated that 0.1% UDCA treatment decreased the expression of YAP in tumors in a concentration-dependent manner when compared with that of the control group (Fig. 6C). In addition, immunohistochemistry data revealed lower expression level of Ki67 and YAP in the UDCA treatment groups when compared with the AOM/DSS group. However, UDCA treatment increased TGR5 expression (Fig. 6D, E). These in vivo results matched with in vitro results, indicating that UDCA suppressed the proliferation of CRC cells by inhibiting YAP and activating TGR5.

**Fig. 7:**
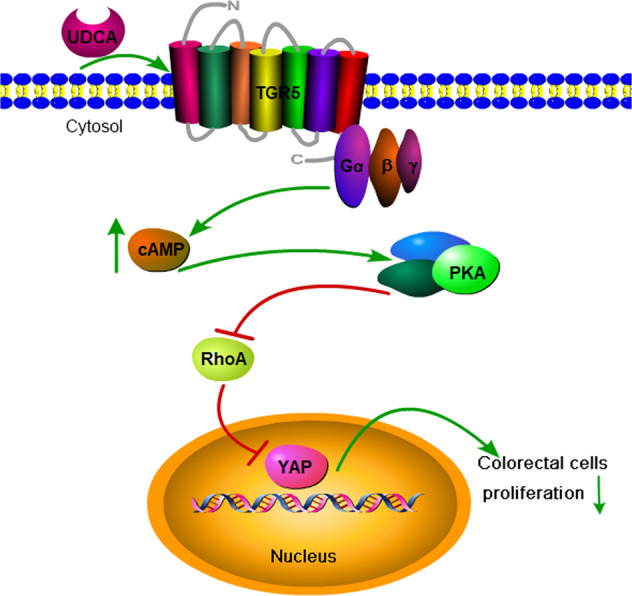
A schematic diagram of how UDCA regulates YAP in HCT116 and SW480 cells. Proposed working model: UDCA stimulates TGR5 on the cell membrane, activated TGR5 increases cellular cAMP levels leading to activation of the cAMP/PKA pathway. Activation of cAMP/PKA pathway inhibits YAP expression by suppressing the RhoA pathway, thereby inhibiting the proliferation and tumor growth of colon cancer cells.

## Discussion

Human bile acid pool mainly consists of primary bile acids CA and CDCA, and secondary bile acids DCA, LCA, TDCA and a small amount of UDCA and TUDCA [10]. The component of the bile acids pool is closely associated with tumorigenesis and tumor growth. The primary BAs, and some secondary bile acids such as DCA and LCA have for long been known as promoters of tumors. In our previous study, we also observed the tumor promotion effect of DCA on CRC (Fig. S3D–E). A previous study reported that LCA stimulated IL-8 expression thereby enhancing the development of CRC [14]. On the other hand, DCA can promote cell proliferation and invasiveness of CRC by activating COX-2, AP-1, and NF-kB signaling [13, 30].

However, accumulating evidence indicates that YAP might promote tumor growth. Abnormal elevation of primary BAs has been found to promote liver tumorigenesis. The primary BAs, CDCA, or CA, act as upstream regulators of YAP via a pathway dependent on IQGAP1 activation. Patients with diverse biliary dysfunctions have high IQGAP1 and nuclear YAP expression [15]. Taurocholate (TCA) can also activate YAP signaling via the G protein-coupled receptor sphingosine 1-phosphate receptor 2 (S1PR2) to promote the growth of esophageal adenocarcinoma [31]. A recent study reported that elevated TDCA levels can stimulate the growth of lymph node-metastatic melanoma via the vitamin D receptor (VDR)-YAP axis [16].

Though UDCA only accounts for a small proportion of the human bile acid pool, it has anti-tumor effects through multiple mechanisms [19, 32, 33]. UDCA inhibited p53 wt colon carcinoma cell proliferation by downregulating c-Myc and the number of cell cycle regulatory molecules, which is consistent with our results [33]. In addition, some studies reported that UDCA prevented DNA damage and activation of oncogenic signaling caused by toxic bile acids such as DCA. UDCA can also block DCA-induced AP-1 and NF-kB activation, and inhibit DCA-induced plasma membrane localization of PKC isoenzymes [30]. This study demonstrates that UDCA inhibits CRC growth by suppressing YAP signaling. Interestingly, the endogenous bile acid tauroursodeoxycholic acid (TUDCA) derived from UDCA has also been reported to promote YAP nuclear exit and degradation, thereby retarding liver over-growth and tumorigenesis [34]. The distinctive effects of UDCA and the primary BAs (or TCA and TDCA) on YAP oncogenic signaling pathways further indicate that UDCA functions as a potent inhibitor of toxic bile acids. The balance of UDCA/TUDCA and toxic bile acids, including the primary BAs, TCA, and TDCA on YAP activity might determine the outcome of tumor prevention or promotion. Moreover, UDCA treatment may shift the balance of bile acid pool [35]. Oral administration of UDCA increased TUDCA and GUDCA levels [36]. Therefore, supplying UDCA in an appropriate dose to patients suffering from bile acid metabolism disorders may be a preventive method for CRC.

TGR5 was the first identified transmembrane G protein-couped bile acid receptor that is ubiquitously distributed throughout the body, especially in the gastrointestinal tract [37, 38]. Conjugated bile acids such as LCA and CDCA mainly interacted with the FXR receptor. However, UDCA acts as a TGR5 receptor agonist instead of a FXR agonist to regulate a series of activities [39, 40]. Moreover, the function of TGR5 depends on the cell and tissue type. The role of TGR5 in the regulation of energy expenditure, glucose metabolism, and bile acid metabolism is well established, but its role in tumors is still controversial. TGR5 is a tumor suppressor in liver cancer, and deficiency of TGR5 promotes chemical-induced tumorigenesis [41]. In addition, TGR5 overexpression in esophageal carcinoma and gastric carcinoma lead to poor prognosis [42, 43]. According to the Oncomine database, TGR5 expression is low in ovarian cancer, breast cancer, colorectal cancer, and lung cancer (Fig. S4A). Colorectal cancer tissues exhibit a lower TGR5 expression level when compared with normal colon and rectum tissue, and decreased TGR5 expression correlates with a poor prognosis after considering the overall survival status (Fig. S4B–C). The results obtained in this study indicated that UDCA treatment increased TGR5 at both mRNA and protein levels in HCT116 and SW480 cells (Fig. 5A, B).

Many studies demonstrated that after stimulating with agonist, GPCR on the cell membrane internalized, and was ubiquitinated and degraded in lysosome [44]. The beta2-adrenoceptor (β2-AR) is a canonical GPCR, the canonical GPCR beta2-adrenoceptor (β2-AR) is internalized, ubiquitinated, and finally degraded after being stimulated with agonist, and also results in increase of cAMP levels [45]. Yang et al. [46] demonstrated that activation of TGR5 with TGR5 agonist resulted in structure change of TGR5. Ligand binding to several mammalian G protein-coupled receptors, such as the PTHR, results in conformational changes and receptor internalization [47]. Therefore, the obvious upregulation of TGR5 in cytoplasm after UDCA treatment may due to internalization and localization change of TGR5 (Fig. 5C). Intestinal stem cells located at the bottom of intestinal crypts generate transit amplifying progenitors, and the transit amplifying progenitors undergo a few cycles of division and finally differentiate into multiple intestinal epithelial cell lineages. Sorrentino et al. [48] indicated that activation of TGR5 in ISCs by BAs promotes regeneration of the intestinal epithelium via avtivating SRC/YAP pathway, which is in consistent with previous view that TGR5 have different functions in different cell and tissue background.

YAP overexpression induced chemotherapy resistance and poses obstacle to the treatment of CRC. UDCA suppressed CRC cells growth through downregulating YAP, and thus may provide new approaches in the treatment of CRC. Downregulation of YAP/TAZ or c-Myc by inhibiting RhoA suppresses cystogenesis in a mouse autosomal dominant polycystic kidney disease model resulting from Pkd1 deficiency [49], and the G-protein-coupled receptor (GPCR) signaling could act through RhoA to regulate the Hippo-YAP pathway. Activation of TGR5 induces smooth muscle relaxation via cAMP/PKA-mediated inhibition of RhoA/Rho kinase pathway [50]. In this study, UDCA, acting as a TGR5 agonist, mainly functions through the cAMP-PKA-RhoA axis to regulate YAP signaling (Fig. 7).

Co-treatment with UDCA and sorafenib or celecoxib demonstrated synergistic anti-tumor effects on HCC or CRC respectively [51, 52]. The UDCA derivate, HS-1183, suppressed cervical carcinoma cells proliferation through activation of JNK and NF-kB, and induced apoptosis in human breast carcinoma cells in a p53-independent pathway [53, 54]. Therefore, further exploration of the anti-tumor mechanism of UDCA derivatives or the combination of UDCA or UDCA derivatives with chemotherapeutic drugs or other anti-tumor drugs is a promising therapeutic strategy.

Taken together, the present study shows that the effect of UDCA on YAP signaling and CRC growth is different from that of primary bile acids and partial secondary bile acids, indicating the importance of maintaining normal intestinal bile acid metabolism in cancer patients. UDCA may therefore be a potential therapeutic intervention for CRC with high TGR5 expression.

## Materials and methods

### Antibodies and reagents

Antibodies plasmids and chemicals used in the study were listed: GAPDH (sc-47724, Santa cruz biotechnology, for western blotting), YAP1 (ab205270, abcam, for western blotting; sc-376830, Santa cruz biotechnology, for Immunofluorescence (IF) and Immunohistochemistry), p-YAP(Ser127) (ab76252, abcam, for western blotting), CYR61 (26689-1-AP, proteintch, for western blotting), c-Myc (ab32072, abcam, for western blotting), CyclinD1 (ab16663, abcam, for western blotting), LaminB1 (PB9611, Boster, for western blotting), RhoA (#2117, CST, for western blotting), ROCK1 (ab134181, abcam, for western blotting), PKA C-ɑ(#4782, CST, for western blotting), TGR5 (ab72608, abcam, for western blotting, for IF and Immunohistochemistry), and Ki67 (#9449, CST, for Immunohistochemistry). Ursodeoxycholic acid was obtained from Target Mol (Shanghai, China), while INT-777 was obtained from Medchemexpress. TGR5 receptor antogonist SBI-115, H89, and SQ22536 were purchased from Selleck, while KT5720 was purchased from Sigma.

### Cell culture and reagents

The human colon cells HCT116 and SW480 were obtained from the Cell Bank of Type Culture Collection of Chinese Academy of Science, Shanghai, China, with mycoplasma contamination detection and STR profiling. The cells were cultured in Dulbecco’s modified Eagle medium containing 10% foetal bovine serum (FBS, Gibio, USA) containing penicillin/streptomycin at 37 °C with a 5% CO_2_ humidified atmosphere.

### Clonogenic assay

The cells were seeded into six-well plates with complete media and then used for the clonogenic assays. After adhering overnight, the cells were treated with the indicated drugs or transfected with plasmids or siRNA before treatment with the indicated drugs. The cells were treated with the indicated drugs for 3–4 days, followed by replacement of growth media with or without drugs every 2 days. After 7–14 days of culture under this condition, the media was discarded, the cells were fixed with 4% paraformaldehyde and then washed with phosphate-buffered saline (PBS) after staining using 0.5% crystal violet for 15 min, followed by photographing.

### Cell viability assays and EdU incorporation assays

MTT assay was used to determine the cell viability. About 5 × 10^3^ cells were seeded per well in 96-well plates, and treated with the indicated drugs after adhering for 24 h in complete medium. Then, the cells were incubated in MTT solution (5 mg/ml) for 4 h, the medium was discarded, and the formazan crystals were dissolved in 150 μl of DMSO followed by measuring of the absorbance at 490 nM. The EdU incorporation assay was then performed using an EdU cell proliferation kit (RiboBio. Guangzhou, China).

### Western blot analysis

For WB analysis, cells were lysed on ice with RIPA buffer (Beyotime) containing protease and phosphatase inhibitors (Roche). The proteins sample (30 μg) were then separated using SDS-PAGE and transferred to a PVDF membrane (Millipore, Bedford, MA, USA) with the BioRad wet transfer system., followed by immunoblotting.

### Small interfering RNA and plasmid transfection

The cells were trypsinised and transferred to six-well pates at ~70–80% confluence. The siRNAs targeting TGR5, PKA Cα, YAP and the negative control were purchased from RiboBio (Guangzhou, China), while the pcDNA3.1, pcDNA3.1YAP, and pcDNARhoA-V14 plasmids used in this study were generated in our laboratory. Transfection of the plasmids and siRNA was performed using Lipofectamine 2000 (Invitrogen/Life Science) according to the manufacturer’s instructions.

### Extraction of nucleoprotein and cytoplasmic protein

The nuclear and cytoplasmic proteins were isolated using a Nuclear and Cytoplasmic Protein Extraction Kit (Sangon Biotech, C510001). The extracted nuclear and cytosolic proteins were then separated using SDS-PAGE and transferred to a PVDF membrane, followed by immunoblotting.

### RNA extraction, reverse transcription, and real-time PCR

Total RNA was extracted using a Cell Total RNA Isolation Kit (Foregene CO.LTD. Chengdu, China) according to the manufacturer’s protocol. Retrotranscription was performed using the Reverse Transcriptase M-MLV (Takara, Japan), while real-time PCR was performed with a SYBR Premix Ex Taq™ kit (Takara, Japan) on the iQ5 Real-Time PCR detection system (Bio Rad, Hercules, USA). primers used in this study were provided in Supplementary Table S1. The expression levels for target genes were normalized to GAPDH and calculated as previous demonstrated [3, 11].

### RhoA activity detection

RhoA activity in the cells was determined using RhoA Pulldown Activation Assay Biochem Kit (Cytoskeleton, Inc, Denver, Colorado, USA). The methods used have been described in a previously published protocol [55].

### Immunofluorescence staining

Cells grown on coverslips were fixed with paraformaldehyde/PBS (4%) for 15 min, and permeabilized in Triton X-100/PBS (0.5%) for 20 min at room temperature. Then, they were blocked with BSA/PBS (3%) for 30 min, and incubated with primary antibodies at 4°C overnight followed by incubating with Alexa Fluor 488-conjugated secondary antibodies (1:1000 dilution) at room temperature for 1 h. After the nuclei were stained with 5 μg/mL DAPI (Invitrogen) for 5 min, the images were captured with a fluorescence microscope (Eclipse 80i, Nikon, Japan) at ×400 magnifications [56].

### Immunohistochemistry

Immunohistochemistry (IHC) was performed using a commercially available immunohistochemical assay kit (Zhongshan Golden Bridge Biotechnology Co., Ltd, Beijing, China). The methods used have been described in a previously published protocol [3, 57].

### Rhodamine-labeled phalloidin staining

HCT116 and SW480 cells were stained using Rhodamine-labeled phalloidin in order to analyze the F-actin cytoskeleton. The cells grown on coverslips were fixed using 3.7% formaldehyde solution for 10 min on ice, and permeabilized with 0.5% Triton X-100 at room temperature for 10 min. They were then stained using Rhodamine-labeled phalloidin (US EVERBRIGHT® INC., Suzhou, China) for 20 min at room temperature, followed by staining with 5 μg/mL DAPI (Invitrogen) for 5 min. The cells were then detected using a fluorescence microscope (Eclipse 80i, Nikon, Japan) at ×400 magnifications.

### cAMP measurements

The whole cell cyclic adenosine monophosphate (cAMP) levels were detected using a Human cAMP ELISA kit (j&l Biological, Shanghai, China) according to the manufacturer’s instructions.

### In vivo experiments

In vivo experiments have been demonstrated before [11]. In all, 5–6-week-old male C57BL/6 mice obtained from Beijing HFK Bioscience. The mice were supplied with food and water ad libitum and maintained under constant temperature and humidity in a 12-h dark/light cycle. All animal experiments were approved by the Experimental Animal Care and Use Committee of University of Tokyo and Tokyo Metropolitan Institute of Gerontology and were performed in accordance with the ARRIVE guidelines.

The mice were divided into five groups: blank group (1), AOM/DSS group (2), AOM/DSS + 0.05% UDCA group (3), AOM/DSS + 0.1% UDCA group (4), and AOM/DSS + 0.2% UDCA group (5) (*n* = 10/per group). Groups two, three, four, and five were administered with a single i.p injection of the mutagen azoxymethane (AOM, Sigma-Aldrich) (10 mg/kg body weight) in combination with three cycles of 1% DSS in drinking water for 7 days, followed by regular drinking water for 14 days. The blank group (group one) were fed with a normal diet, while groups three, four, and five were fed with a diet containing 0.05%, 0.1%, and 0.2% UDCA, respectively. All the in vivo experiments were performed according to the institute guidelines and approved by the Animal Ethics Committee of the China Institute of Science.

### Statistical analysis

The qualification of WB assay and clonogenic assay was performed using the Image J. All the statistical calculations were performed using the GraphPad Prism 5. The data were expressed as mean ± S.D., and analyzed with a two-tailed Student’s *t* test. Statistical significance was defined as *P*-value of < 0.05 (*), < 0.01 (**), and < 0.001 (***). No statistical methods were used to predetermine sample size, and all experiments were performed using at least three biological replicates.

## Supplementary information

Supplementary Figure1

supplementary Figure2

Supplementary Figure 3

Supplementary Figure 4

Supplementary Tables
