# Supplementary figures and images for: Ursodeoxycholic acid suppresses the malignant progression of colorectal cancer through TGR5-YAP axis
Source: Cell Death Discov. 2021 Aug 7;7:207. doi: 10.1038/s41420-021-00589-8 (PMC8349355; doi:10.1038/s41420-021-00589-8)

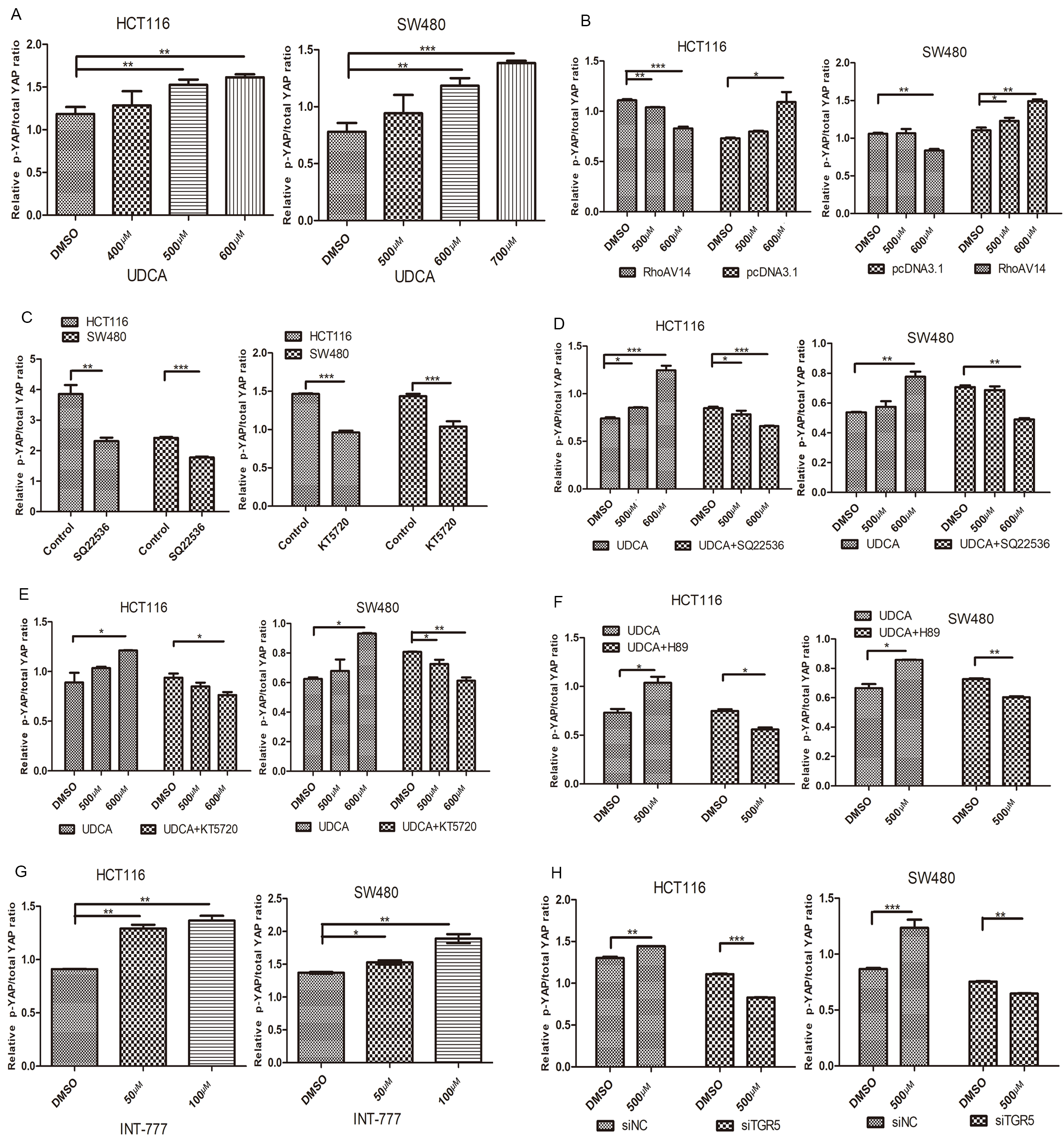

Supplement: Supplementary file 1 — Supplementary Figure1 [file 41420_2021_589_MOESM1_ESM.tif]

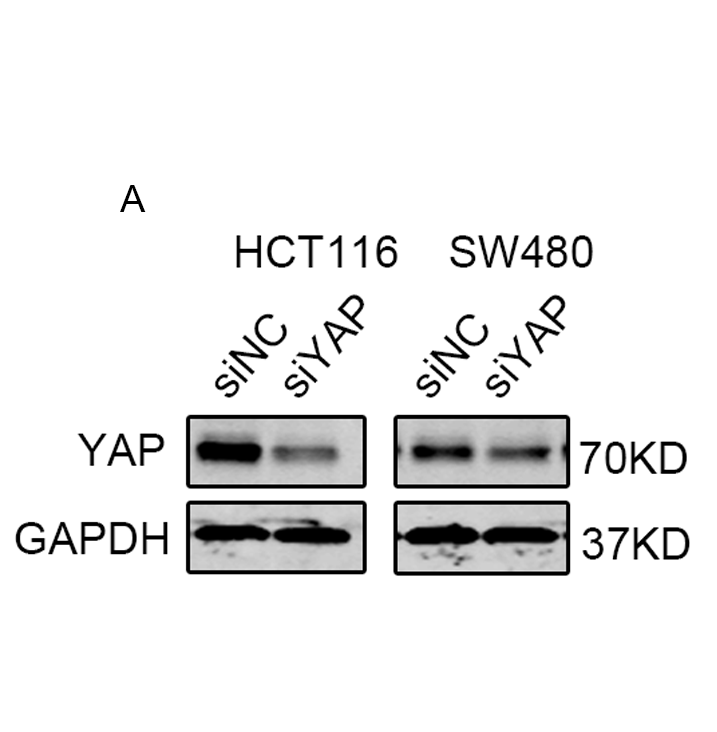

Supplement: Supplementary file 2 — supplementary Figure2 [file 41420_2021_589_MOESM2_ESM.tif]

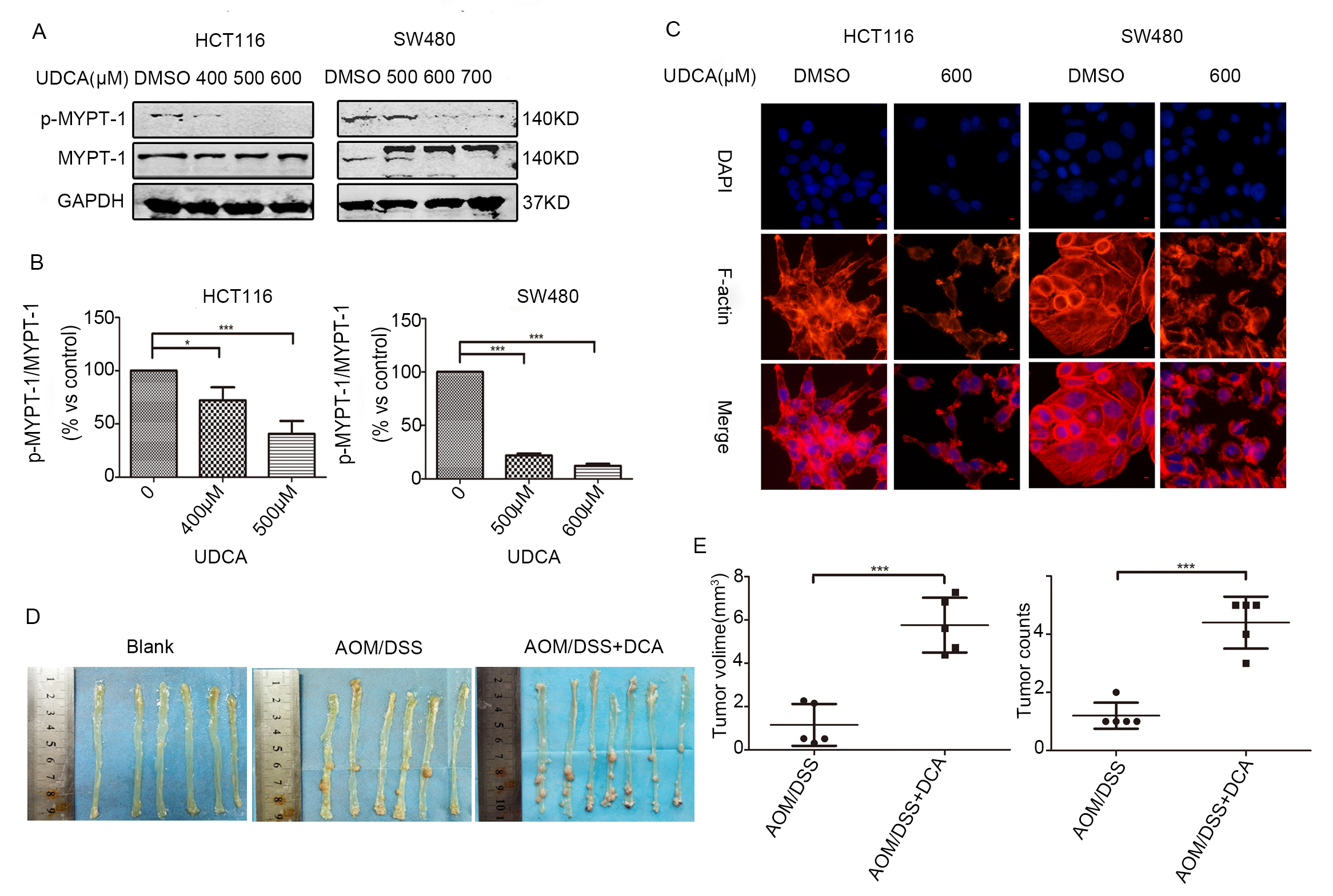

Supplement: Supplementary file 3 — Supplementary Figure 3 [file 41420_2021_589_MOESM3_ESM.tif]

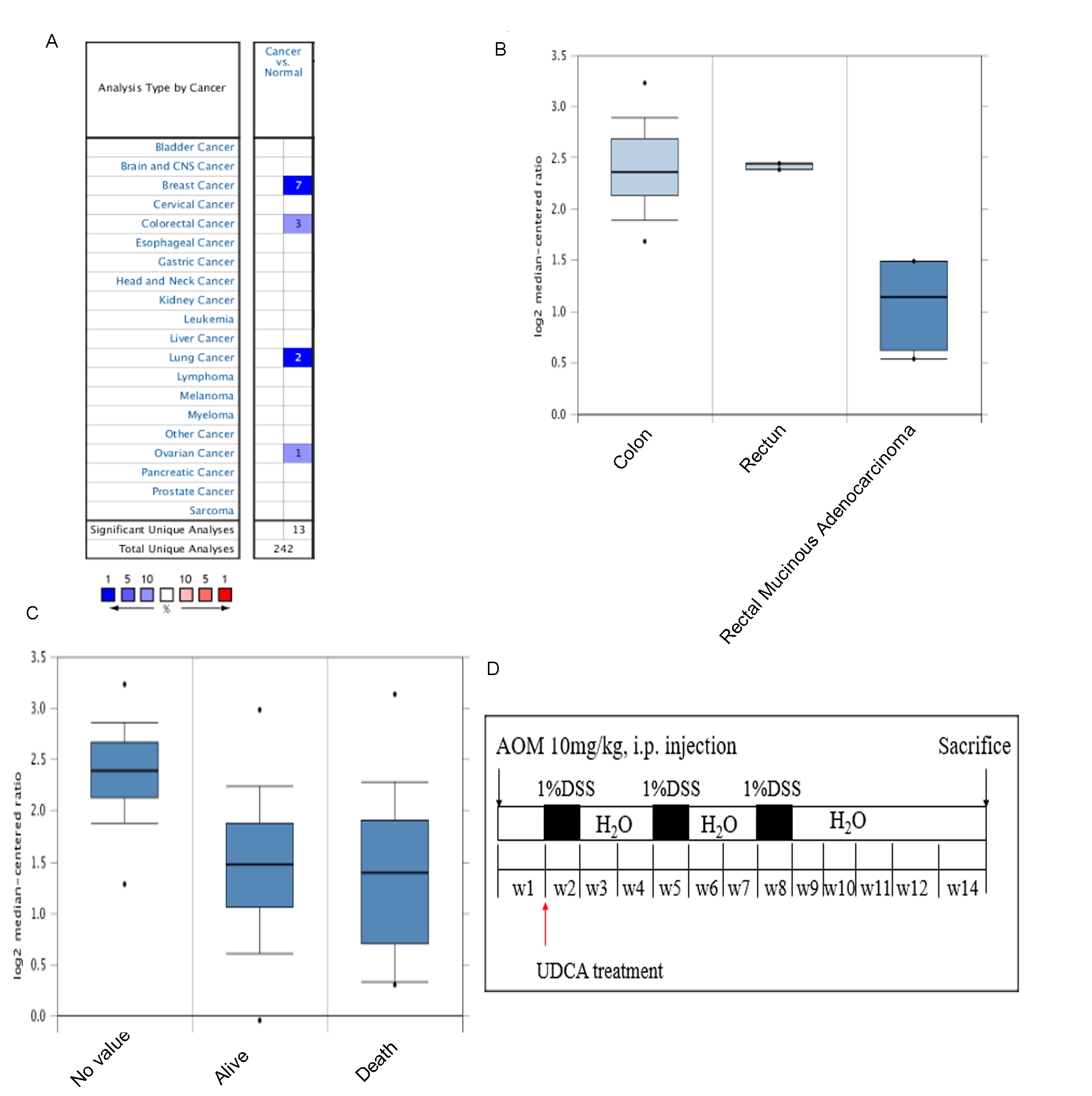

Supplement: Supplementary file 4 — Supplementary Figure 4 [file 41420_2021_589_MOESM4_ESM.tif]
