# Supplementary material for: Ursodeoxycholic acid suppresses the malignant progression of colorectal cancer through TGR5-YAP axis
Source: Cell Death Discov. 2021 Aug 7;7:207. doi: 10.1038/s41420-021-00589-8 (PMC8349355; doi:10.1038/s41420-021-00589-8)
Supplement: Supplementary file 5 — Supplementary Tables [file 41420_2021_589_MOESM5_ESM.docx]

Supplementary Table S1. Primer list for real-time PCR

| Gene | Forward | Reverse |
| --- | --- | --- |
| GAPDH | 5′-AGAAGGCTGGGGCTCATTTG-3′ | 5′-AGGGGCCATCCACAGTCTTC-3′ |
| CYR61 | 5′-AGCCTCGCATCCTATACAACC-3′ | 5′-TTCTTTCACAAGGCGGCACTC-3′ |
| RhoA | 5′-ACACACCAGGCGCTAATTCA-3′ | 5′-CCCCAGAGCTATGCCAACAA-3′ |
| TGR5 | 5′-GCTGCTTCTTCCTGAGCCTA-3′ | 5′-GTTGGGAGCCAAGTAGACGA-3′ |
| YAP | 5′-AGCTGCCCGACTCCTTCTTC-3′ | 5′-GAGGAATGAGCTCGAACATGC-3′ |
